# Diclofenac Administration after Physical Training Blunts Adaptations of Peripheral Systems and Leads to Losses in Exercise Performance: In Vivo and In Silico Analyses

**DOI:** 10.3390/antiox10081246

**Published:** 2021-08-04

**Authors:** Rômulo Pillon Barcelos, Frederico Diniz Lima, Aline Alves Courtes, Ingrid Kich da Silva, Jose Eduardo Vargas, Luiz Fernando Freire Royes, Cristiano Trindade, Javier González-Gallego, Félix Alexandre Antunes Soares

**Affiliations:** 1 Programa de Pós-Graduação em Bioquímica Toxicológica, Centro de Ciências Naturais e Exatas, Departamento de Bioquímica e Biologia Molecular, Universidade Federal de Santa Maria, Santa Maria 97105-900, Brazil; alinecourtes@bol.com.br (A.A.C.); ingrid.ksilva@gmail.com (I.K.d.S.); felix@ufsm.br (F.A.A.S.); 2 Programa de Pós-Graduação em Bioexperimentação (PPGBioexp), Universidade de Passo Fundo (UPF), BR 285, Passo Fundo 99052-900, Brazil; 3 Laboratório de Bioquímica do Exercício, Centro de Educação Física e Desportos, Universidade Federal de Santa Maria, Santa Maria 97105-900, Brazil; profred@gmail.com (F.D.L.); nandoroyes@yahoo.com.br (L.F.F.R.); 4 Laborátorio de Biologia Molecular, Instituto de Ciências Biológicas (ICB), Universidade de Passo Fundo (UPF), Passo Fundo 99052-900, Brazil; josevargas@upf.br; 5 Hospital de Clínicas de Porto Alegre, Programa de Pós-Graduação Ciências em Gastroenterologia e Hepatologia, Universidade Federal do Rio Grande do Sul (UFRGS), Porto Alegre 90035-003, Brazil; 6 Facultad de Ciencias Básicas y Biomédicas, Universidad Simón Bolívar, Barranquilla 080002, Colombia; 7 Institute of Biomedicine (IBIOMED) and Centro de Investigación Biomédica en Red de Enfermedades Hepáticas y Digestivas (CIBERehd), University of León, 24071 León, Spain; jgonga@unileon.es

**Keywords:** NSAIDs, physical training, rat, diclofenac, inflammation, oxidative stress, systems biology, systems pharmacology, adaptation

## Abstract

Recovery in athletes is hampered by soreness and fatigue. Consequently, nonsteroidal anti-inflammatory drugs are used as an effective strategy to maintain high performance. However, impact of these drugs on adaptations induced by training remains unknown. This study assessed the effects of diclofenac administration (10 mg/kg/day) on rats subjected to an exhaustive test, after six weeks of swimming training. Over the course of 10 days, three repeated swimming bouts were performed, and diclofenac or saline were administered once a day. Trained animals exhibited higher muscle citrate synthase and lower plasma creatinine kinase activities as compared to sedentary animals, wherein diclofenac had no impact. Training increased time to exhaustion, however, diclofenac blunted this effect. It also impaired the increase in plasma and liver interleukin-6 levels. The trained group exhibited augmented catalase, glutathione peroxidase, and glutathione reductase activities, and a higher ratio of reduced-to-oxidized glutathione in the liver. However, diclofenac treatment blunted all these effects. Systems biology analysis revealed a close relationship between diclofenac and liver catalase. These results confirmed that regular exercise induces inflammation and oxidative stress, which are crucial for tissue adaptations. Altogether, diclofenac treatment might be helpful in preventing pain and inflammation, but its use severely affects performance and tissue adaptation.

## 1. Introduction

A regular exercise regime is well-known to confer significant health benefits. In particular, it reduces the risk of severe diseases, including type 2 diabetes, cardiovascular diseases, cancer, or neurodegeneration. In general, regular exercising plays a pivotal role in maintaining and improving organism functions, and also influences growth and life expectancy [1,2]. However, acute exercise may lead to inflammation and a redox imbalance in the body, which further induces the activation of various signaling pathways in tissues, such as muscle and the liver. Despite their possible role in incurring damaging effects, inflammation and oxidative stress are also known to be involved in the exercise-induced adaptation process [3]. Thus, acute and exhausting exercises induce an increase in the levels of various circulating cytokines and chemokines, including interleukin-1 (IL-1), IL-6, and tumor necrosis factor-α (TNF-α), which could either harm the organism or may assist in tissue adaptation after a period of recovery [4]. However, during sports competitions, athletes are required to perform the tasks within small intervals, sometimes days or maybe a few hours, and thus they do not get ample time to ensure sufficient recovery. As a consequence, nonsteroidal anti-inflammatory drugs (NSAIDs) are commonly used to decrease the acute inflammatory response, soreness, and pain during such competitions. During the last decade, diclofenac was one of the most commonly prescribed over-the-counter NSAIDs [5].

Similar to classic NSAIDs, diclofenac also acts via inhibition of cyclooxygenase enzyme activities that decreases the production of prostaglandin endoperoxides, and thus relieves inflammation and pain. In particular, diclofenac is prescribed during sports competitions to reduce pain-related symptoms, while ensuring fast recovery from previous strenuous exercises and enabling constant preparation for the next competitive task [6,7]. Recent studies from our laboratory demonstrated that diclofenac pretreatment decreased the expression and protein content of several molecules involved in inflammatory pathways, including TNF-α, toll-like receptor-4, or p65 nuclear factor kappa B (NF-κB) subunit, after eccentric exercise bouts in rat muscle [8] and liver [9]. Such pretreatment might assist in fast recovery; however, this may also lead to non-adapted tissues, thereby limiting some of the beneficial effects of training.

Regular physical training involves a different set of adaptation mechanisms as compared to acute exercise [10,11]. However, no information is currently available regarding the association between NSAIDs and the effects of training on plasma or peripheral organs, such as the liver. In recent years, systems pharmacology has emerged as a holistic approach in pharmacology that provides novel insights to unravel molecular mechanisms involved in the action of drugs [12,13,14]. In particular, the use of in silico analysis, based on this approach, might aid in the identification of molecular targets of diclofenac that mediate the physiological adaptation to exercise. Thus, this study aimed to investigate the overall impact of diclofenac on the response to acute bouts of exercise after a 6-week training period. The study combined the results of molecular analysis, obtained from an in vivo study, with systems pharmacology predictions, and identified changes in the redox and inflammatory status.

## 2. Materials and Methods

### 2.1. Animals and Ethics Statement

The present study utilized 3-month-old male Wistar rats, weighing 270 ± 25 g, obtained from our own breeding colony (UFSM), for in vivo experiments. The animals were maintained in an air-conditioned room at 25 ± 1 °C with 55% relative humidity and 12/12 h light/dark cycle. The animals were fed standard laboratory chow and had free access to water. All experimental procedures were conducted in accordance with the National and International Legislations, Brazilian College of Animal Experimentation (COBEA) and the US Public Health Service’s Policy on Human Care and Use of Laboratory Animals-PHS Policy. The experimental protocols included in the study were approved by the local Ethics Committee.

### 2.2. Experimental Design and Diclofenac Administration

One week prior to swimming training, the animals were initially exposed to water conditions for acclimatization, with the aim to reduce stress and make them acquainted to the exercise environment. In particular, the water adaptation process involved keeping the animals in shallow water (5 cm in depth) at 31 ± 1 °C from 9 am to 11 am, for one week. Following this, the animals were randomly divided into two groups, training (*n* = 21) and sedentary (*n* = 21) groups. The training group was subjected to a 6-week swimming training program with bodyweight overload of 5% of body weight, 60 min per day for 6 weeks [15,16]. The tank used for training was 80 cm in length, 50 cm in width, and 90 cm deep. The swimming training was always performed with water temperature at 31 ± 1 °C, for a duration of 2 h (10–12 am). In comparison, rats in the sedentary group were placed in a separate but similar tank, with shallow water (5 cm), at the same temperature for 30 min, 5 days a week, and without any back overload. At the end of each training schedule, rats were towel-dried and returned to their respective cages.

After completion of the training process, both training and sedentary groups were sub-divided into saline or diclofenac administration groups. Thus, the study involved a total of four groups, namely group I, Sed-Sal, sedentary untrained animals, treated with saline (vehicle; *n* = 10); group II, Sed-Diclo, sedentary untrained animals, treated with diclofenac 10 mg/kg/day (*n* = 10); group III, Exe-Sal, 6-week swimming trained animals, treated with saline (vehicle) (*n* = 11); and group IV, Exe-Diclo, 6-week swimming trained animals, treated with diclofenac 10 mg/kg/day (*n* = 11). Following a previously established method to simulate the use of diclofenac during competition tasks, the swimming groups were subjected to three exhaustive swimming bouts, where each bout was separated by a period of 72 h [9].

Diclofenac (Sigma-Aldrich, St. Louis, MO, USA) was administered orally by intragastric gavage, at a dose of 10 mg/kg body weight (in saline) for 9 days. This dose was used in previous studies [9,17] and is and is below those known to induce hepatotoxicity [18,19]. Prior to the first exhaustion bout, the animals were pretreated for three days, and the treatment was continued till the last day of the protocol. The non-treated animals received saline during the same treatment period.

**Figure 1:**
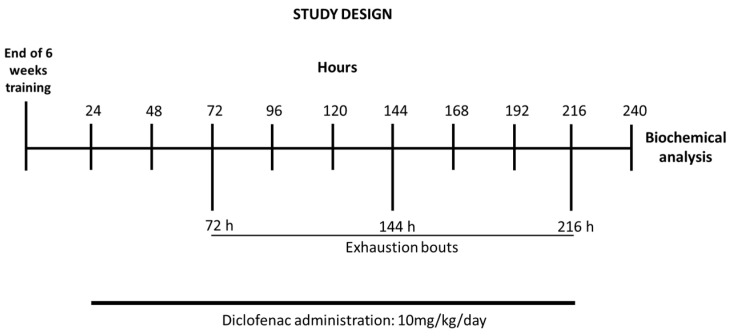
Timeline of the exercise training schedule, exhaustive protocol test, and data collection.

Following the last bout, rats were sacrificed by decapitation. After exsanguination, the liver was immediately excised, freeze-clamped between aluminum tongs (precooled with liquid nitrogen) and stored at −80 °C for biochemical assays. Schematics of the study design are shown in Figure 1.

### 2.3. Biochemical Plasma Parameters

Plasma levels of glucose, creatinine kinase (CK), aspartate transaminase (AST), alanine transaminase (ALT), triglycerides (TG), total cholesterol (TC), uric acid (UA), high-density lipoprotein (HDL), albumin, and alkaline phosphatase (ALP) were estimated using commercially available standard biological kits (Labtest, Lagoa Santa, Brazil).

### 2.4. Quantification of Extracellular Double-Stranded DNA (dsDNA)

To assess cell damage/death, the presence of dsDNA fragments in the plasma was measured using PicoGreen^®^ fluorescent assay [20]. The test was performed in 96-well fluorescence microplates using Quant-iT™ PicoGreen^®^ kit (Invitrogen, Eugene, OR, USA), as per the manufacturer’s instructions. The fluorescence measurements were recorded using a RF-5301 Shimadzu spectrofluorometer, wherein fluorescence emissions for PicoGreen^®^ alone (blank) and PicoGreen^®^ with DNA were recorded at room temperature (25 °C), with excitation and emission maxima of 480 nm and 520 nm, respectively. Results were expressed in terms of fluorescence of dsDNA.

### 2.5. IL-6 Levels

The levels of IL-6 in the liver and plasma were measured by enzyme linked immunosorbent assay, using a commercial kit (IL-6, eBIOSCIENCE^®^, San Diego, CA, USA). Briefly, 96-well microplates were sensitized with the primary antibody at room temperature for 30 min. Following this, the samples were added and incubated at 37 °C for 30 min. Further, the samples were washed as per the manufacturer’s instructions. Post washing, a peroxidase-conjugated secondary antibody was added to each well. The concentration of the cytokines was determined in terms of the conversion of substrate, which was measured spectrophotometrically using a microplate reader at a wavelength of 480 nm.

### 2.6. Catalase (CAT) Activity

The enzymatic activity of CAT was determined according to the method proposed by Aebi (1984) [21]. The CAT enzyme activity was measured in terms of the degradation of H_2_O_2_, where the absorbance of the colored reaction cocktail was measured at 240 nm. The resulting data were corrected by protein content and expressed as the percentage of control.

### 2.7. Glutathione Peroxidase (GPx) Activity

The GPx activity was determined in terms of conversion of NADPH to NADP, which was measured spectrophotometrically at 340 nm [22]. The enzymatic reaction was initiated by the addition of H_2_O_2_ to a final concentration of 0.4 mM. The reaction was performed at 30 °C for 2 min, and GPx activity was determined using a molar extinction coefficient of 6220 M^−1^ cm^−1^. The GPx activity of the samples was expressed as a percentage of control.

### 2.8. Glutathione Reductase (GR) Activity

To determine GR activity, the enzymatic reaction was initiated by the addition of 20 mM glutathione (oxidized). The reaction mixture was incubated at 30 °C for 2 min and the measurements were performed at 340 nm [23]. The GR activity was determined using the molar extinction coefficient of 6220 M^−1^ cm^−1^. The resulting sample readings were corrected by the protein content and expressed as a percentage of control.

### 2.9. Reduced (GSH) and Oxidized (GSSG) Glutathione

The levels of GSH and GSSG in the liver were measured as per the previously established method [24]. Fluorescent signals were recorded using a spectrofluorometer, with excitation and emission maxima of 350 nm and 420 nm, respectively, and compared with linear standard curves for GSH and GSSG. The results were expressed as GSH/GSSG ratio.

### 2.10. Citrate Synthase (CS) Activity

Activity of CS in mixed gastrocnemius muscle and liver tissues was determined spectrophotometrically, according to the previously established method [25]. The tissue samples were homogenized and CS activity was measured in terms of the condensation of acetyl-CoA and oxaloacetate. The reaction was performed at 25 °C and the concentration of resulting complex was determined at 412 nm. The CS activity was expressed as percentage of control.

### 2.11. Protein Determination

The protein content was determined using bovine serum albumin (BSA) (1 mg/mL) as standard, according to previously described protocol [26].

### 2.12. Statistical Analyses

All the datasets were subjected to statistical analyses using GraphPad Prism v.8.01 (Graphpad Inc., La Jolla, CA, USA). For continuous data showing normal distribution, datasets were analyzed using two-way analysis of variance, with 2 (diclofenac and saline treatments) × 2 (sedentary and trained) variables. This was followed by Sidak’s post hoc test, and *p*-values < 0.05 were considered to be statistically significant. For each experimental set up, all data sets were expressed as means ± SEM.

### 2.13. In Silico Analysis

The system biology approach combined with in silico analysis offers an efficient computational tool that can better explain interaction of bioactive molecules, such as diclofenac, with the signal molecules of various cellular pathways. In order to obtain curated genes/proteins, MitoCarta3.0 inventory was used, with a cut-off ≥2 [27]. Subsequently, a chemical-protein (CP)–protein–protein interaction (PPI) (CP-PPI) network was proposed for *Rattus norvergicus*, based on the genes and inflammatory cytokines obtained from MitoCarta3.0 inventory, using a plug-in STITCH (stringApp 1.6) from Cytoscape 3.8.2 [28]. STITCH pipeline includes high-throughput experiments data, manually curated datasets, and results from multiple prediction methods into a single global network of protein–protein and protein–chemical interactions. In particular, STITCH tool allowed the visualization of the connections (edge) among different nodes (genes or proteins) and diclofenac-nodes, where each edge possessed a degree of confidence between 0 and 1.0. Here 0 represented lowest confidence and 1.0 represented highest confidence. For further analysis, nodes without any connections were excluded.

Further, MCODE 2.0.0 was used to predict clusters from CP-PPI network in which the existing nodes were modulated by diclofenac [29]. In particular, this plug-in predicts the occurrence of densely connected regions in a network on the basis of a graph-theoretic clustering algorithm. Following this, enrichment network analysis was performed using Cytoscape ClueGO 2.5.7 plug-in [30]. Gene ontology (GO) and Kyoto Encyclopedia of Genes and Genomes (KEGG) pathways are used to elucidate and describe molecular functions and biological and signal processes of genes. Updated annotations from GO and KEGG pathways were calculated using the hypergeometric test and a significant *p*-value threshold of 0.001 (FDR adjusted) was used for analysis.

For further analysis of the CP-PPI network, Cytoscape plug-in, CentiScaPe 3.2.1, was used, and degree and betweenness parameters were considered [30]. In general, degree indicates the number of adjacent nodes connected to a unique node, while betweenness refers to the number of shortest paths between two nodes that pass through a targeted node [31]. We finally investigated if there were hub-bottleneck (H-B) nodes possessing a high value of degree and betweenness which could be topologically crucial to the network structure.

## 3. Results

### 3.1. Time to Exhaustion

**Figure 2:**
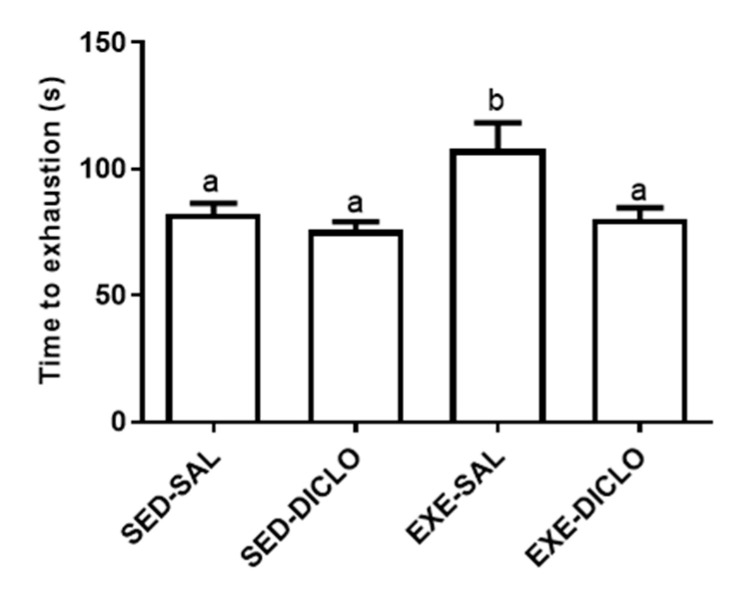
Effect of training and diclofenac treatment on time to exhaustion. At the end of six weeks of training period, trained and sedentary groups were divided into two groups each: sedentary-saline (Sed-Sal), sedentary-diclofenac (Sed-Diclo), exercise-saline (Exe-Sal), and exercise-diclofenac (Exe-Diclo) groups. Following this, rats were subjected to three exhaustive swimming bouts, separated by a period of 72 h. For each group, results represent the mean of the three bouts. Values are expressed as mean ± SEM. Means for a variable with superscripts without a common letter differed significantly (*p* < 0.05).

The present study evaluated the effects of administration of diclofenac (10 mg/kg/day) on the response to acute bouts of exercise in rats subjected to six weeks of swimming training. The time to exhaustion was calculated as a mean of the three exhaustion bouts tests, following six weeks of training program. As shown in Figure 2, animals from the Exe-Sal group exhibited an increased time to exhaustion as compared to the Sed-Sal group. However, no significant differences were reported between the Exe-Diclo and sedentary control groups. In fact, Exe-Diclo group was characterized by a lower time to exhaustion as compared to the Exe-Sal group, negating the effect of training on this parameter.

### 3.2. Physical Training Markers

**Figure 3:**
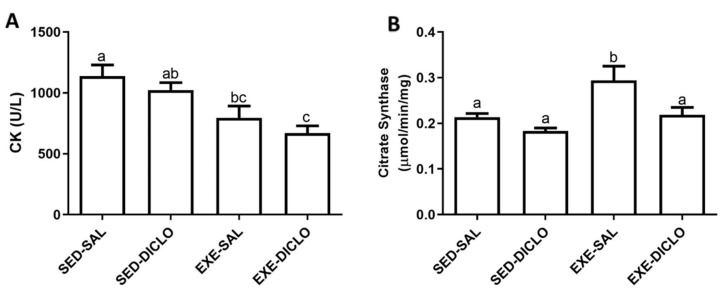
Effect of training and diclofenac treatment on the enzymatic activities of (**A**) plasma creatine kinase (CK) and (**B**) muscle citrate synthase (CS). Values are expressed as mean ± SEM. Means for a variable with superscripts without a common letter differed significantly (*p* < 0.05).

The present study evaluated the effect of training and diclofenac treatment on the activities of plasma CK and muscle CS enzymes. In general, CK and CS act as markers of muscle damage and metabolic activity, respectively. As shown in Figure 3, lower CK activity was recorded in plasma samples obtained from trained groups (Exe-Sal and Exe-Diclo) as compared to the Sed-Sal group (Figure 3A). In comparison to this, the evaluation of muscle CS activity showed that Exe-Sal group was characterized by a higher CS activity as compared to the control group. No significant differences were recorded between CS activity of the Exe-Diclo and control groups; however, the CS activity of Exe-Diclo group was significantly lower as compared to the Exe-Sal group (Figure 3B).

### 3.3. Plasma Biochemical Parameters

Both trained groups, Exe-Sal and Exe-Diclo, showed significantly lower triglycerides levels as compared to the control group, Sed-Sal (Table 1). Interestingly, no significant effect of exercise or diclofenac treatment was reported on glucose concentration, enzyme activities, or dsDNA content in the plasma samples.

Values are expressed as mean ± SEM. Means for a variable with superscripts without a common letter differed significantly (*p* < 0.05). ALT, alanine transaminase; AST, aspartate transaminase; ALP, alkaline phosphatase; dsDNA, double-stranded DNA.

### 3.4. IL-6 Levels

**Figure 4:**
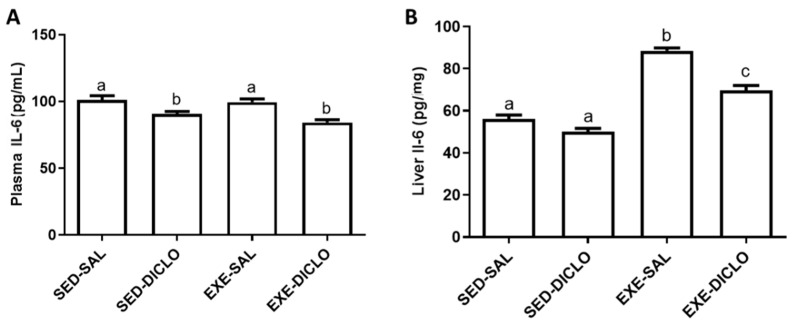
Effect of training and diclofenac treatment on the levels of interleukin-6 (IL-6) in (**A**) plasma and (**B**) liver. Values are expressed as mean ± SEM. Means for a variable with superscripts without a common letter differed significantly (*p* < 0.05).

The effects of training and diclofenac administration on IL-6 levels in the plasma and liver are shown in Figure 4. As shown in Figure 4A, diclofenac administration resulted in a decrease in the plasma IL-6 levels. Interestingly, higher levels of IL-6 were recorded in the liver of Exe-Sal as compared to the control group; however, diclofenac treatment (Exe-Diclo group) resulted in a significant reduction in these effects (Figure 4B).

### 3.5. In Silico Analysis and Redox Balance

**Figure 5:**
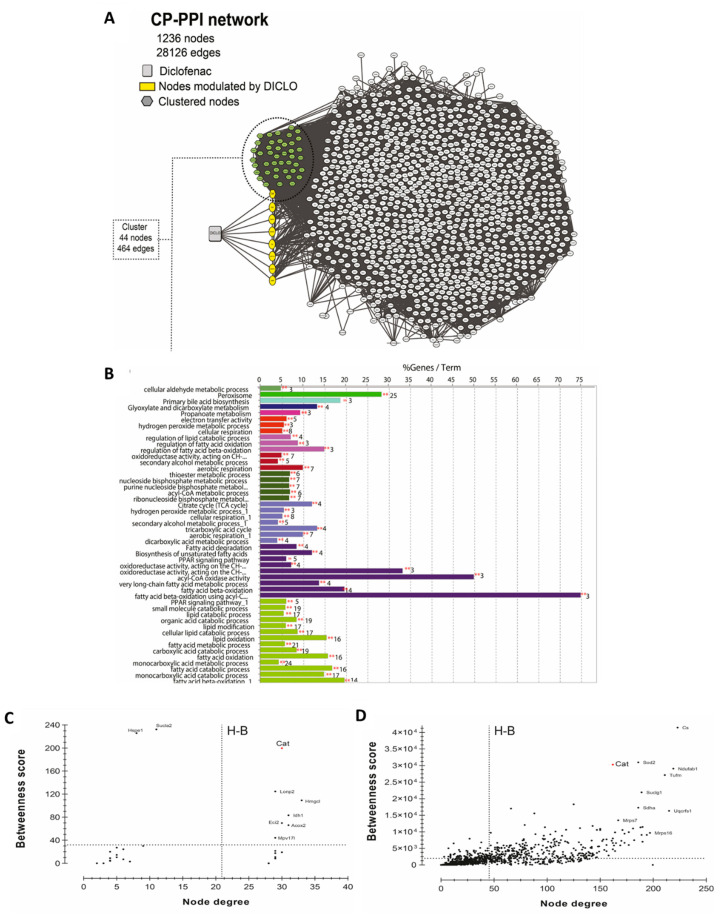
Systems biology analysis for the prediction of diclofenac interactors. (**A**) CP-PPI network. Significant module from this network was obtained using MCODE 2.0.0. Here, clustered genes/proteins are represented by nodes of different colors and diclofenac is represented by a square shape node. (**B**) GO/KEGG analyses were performed using ClueGO algorithms. Centrality analysis of (**C**) cluster and (**D**) CP-PPI network. Here, dashed lines represent the threshold value calculated for each centrality. Genes/proteins are represented by black dots, and catalase is denoted by a red dot. Only node scores above the network average are indicated; Hub-bottleneck (H-B).

To generate the network, 1329 curated genes and proteins were used as initial input in the STITCH plug-in. As shown in Figure 5A, a CP-PPI network, comprising of 1236 nodes and 28,126 edges was generated. Following this, two topological analyses were applied, clustering and GO/KEGG enrichments. The use of MCODE identified the occurrence of only an enriched cluster in the CP-PPI network that was composed of 44 nodes, where a node was found to be directly modulated by diclofenac. This unique node modulated by diclofenac was identified to be the CAT enzyme.

To get better insights into the biological putative function or pathways involved within this cluster, enrichment analysis was performed using ClueGO software. Enrichment analysis is a method to identify classes of genes or proteins over-represented in a large set of genes or proteins and may associate with a specific cellular function into an annotation. As shown in Figure 5B, 49 GO terms were found to be significantly enriched, of which four groups possessed CAT. According to enrichment analysis, the main GO categories that were identified included peroxisome (corrected *p*-value = 4.28 × 10^−47^), glyoxylate and dicarboxylate metabolism (corrected *p*-value = 3.82 × 10^−6^), secondary alcohol metabolic process (corrected *p*-value = 2.01 × 10^−12^), cellular respiration (corrected *p*-value = 8.33 × 10^−11^), and hydrogen peroxide metabolic process (corrected *p*-value = 9.05 × 10^−9^).

To investigate if CAT acted as a H-B node in the cluster or within the whole CP-PPI network, topological centrality analysis was performed. As shown in Figure 5C,D, CAT was identified as an H-B in the cluster and within all CP-PPI networks. These results suggested that CAT is a key node and pharmacological modulations on it can promote pathway-network perturbations.

**Figure 6:**
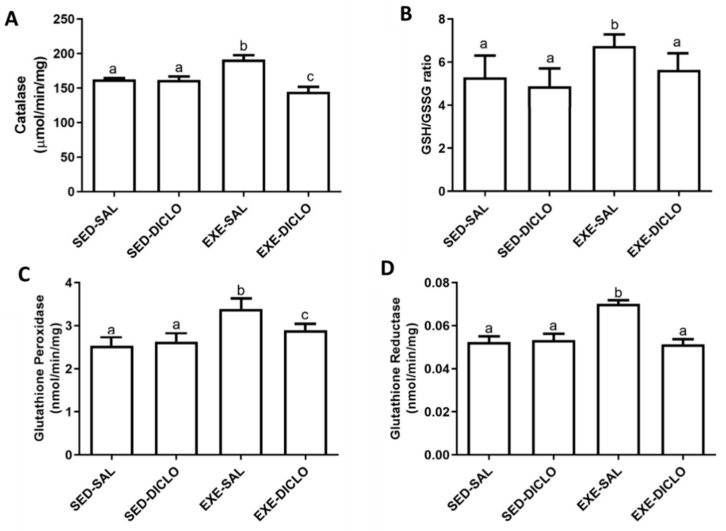
Effect of training and diclofenac treatment on redox parameters, (**A**) catalase (CAT) activity, (**B**) reduced to oxidized glutathione ratio (GSH/GSSG), (**C**) glutathione peroxidase (GPx) activity, and (**D**) glutathione reductase (GR) activity. Values are expressed as mean ± SEM. Means for a variable with superscripts without a common letter differed significantly (*p* < 0.05).

Further, to validate the impact of diclofenac on CAT, the activity of CAT enzyme was measured in the experimental model used in the present study (Figure 6A). Liver CAT enzyme activity was found to be significantly higher in the Exe-Sal group as compared to its control, whereas the Exe-Diclo group showed lower CAT activity as compared to both Sed-Sal and Exe-Sal groups (Figure 6A).

In addition to this, diclofenac treatment introduced significant alternations in the levels of other components of the redox cycle as well. As shown in Figure 6B, an increased ratio of GSH/GSSG was reported in the Exe-Sal group as compared to the control, and this effect was blunted by diclofenac treatment (Exe-Diclo). Interestingly, augmented liver GPx activity was observed in the Exe-Sal group as compared to the control, and diclofenac treatment induced reduction in these values (Figure 6C). In particular, these values were significantly different from the Sed-Sal and Exe-Sal groups (Figure 6C). Similar results were reported for glutathione reductase (GR) activity (Figure 6D), where an increased GR activity was observed in the Exe-Sal group as compared to control, which was reversed by diclofenac treatment.

## 4. Discussion

The present study explored the impact of oral administration of diclofenac (10 mg/kg body weight) on the response to acute bouts of exercise in rats, after six weeks of training period. In particular, the rats subjected to the 6-week swimming training program displayed reduced plasma CK levels and augmented muscle CS activity, after an exhaustion test. These results were in accordance with the findings of previous studies that reported favorable adaptations involving the aerobic metabolism in response to regular training [32,33]. Additionally, these results might also explain the observations regarding the increased time to reach exhaustion [34,35]. However, a 10-day diclofenac treatment, after training, blunted most of these changes, and a decreased time-to-exhaustion performance and reduced muscle CS enzyme activity was reported in groups receiving diclofenac. In concordance with previous findings, physical exercise decreased the plasma content of triglycerides. Interestingly, this effect was blocked by diclofenac treatment. More importantly, diclofenac treatment did not induce any significant changes in enzymatic activities or dsDNA content in plasma, indicating absence of any toxic effects of the drug.

Our results, particularly the inflammatory and redox status in the plasma and liver, highlighted that the training program activated specific tissue adaptive responses in rats. However, administration of diclofenac blunted most of these tissue adaptations. This is particularly important as no previous studies reported such a loss in tissue adaptation after regular exercise by the administration of a commonly used drug. A similar blocking of adaptation effects during training was previously reported with regard to antioxidant supplementation. In particular, antioxidants act as ROS and RNS scavengers, and thus produce a deleterious impact on muscle response to stress, leading to lower tissue adaptation after high-intensity exercises. This adaptation might be attributed to the fact that ROS and RNS act as signaling molecules, which are involved in enhancing protection against physical stress [36,37]. Several previous studies also reported that some drugs or supplements, such as caffeine and other NSAIDs, might impede long-term adaptations to physical exercise by preventing oxidative stress and inflammatory damage [38,39]. Thus, the performance losses observed in the present study might be related to reduction in tissue adaptations after diclofenac treatment.

Apart from this, the previous studies from our laboratory demonstrated that diclofenac pretreatment decreased the expression and content of several cytokines and proteins related to inflammatory pathways, such as TNF-α, toll-like receptor-4, and p65 NF-κB subunit, after eccentric exercise bouts in rat muscle [8] and liver [9,17]. During sports competitions, or similar tasks, athletes need constant and increased muscle efforts, thereby making use of NSAID strategy quite common and useful. It ensures a fast recovery to the athlete that further allows them to participate in another competitive task, taking place within short durations of time. However, use of such strategies should be avoided for long-term adaptation as it may lead to a non-adapted tissue, blunting the beneficial effects of physical training, as reported in the present study.

In the present research, the trained group exhibited an increased level of IL-6, both in the plasma and liver. Post exercise training, such an increase in IL-6 production, is indeed a part of the adaptive mechanism, which provides a challenging environment to tissues and triggers them to respond to this inflammation process [8,38]. IL-6 is known to activate the immune system and improve glucose tolerance and insulin sensitivity after physical exercise [39,40]. Additionally, IL-6 has been previously shown to stimulate the release of IL-1Ra and IL-10 in human mononuclear cells [41], and to inhibit the transcription of IL-1 and TNF-α [42]. Similar results for IL-6 were reported in the present case. In particular, these mechanisms are triggered by several factors, including training frequency, intensity, volume, and the experimental model [43]. Here, diclofenac treatment blunted this response, and lower levels of IL-6 were reported in the plasma and liver of Exe-Diclo animals as compared to the trained but non-treated animals (Exe-Sal group). The anti-inflammatory effects of diclofenac are widely reported, and these effects might be responsible for the observed reduction in the tissue response mechanisms, which further resulted in a less adapted organism.

Several authors reported a well-established relationship between physical exercise, inflammation, and oxidative stress [44,45]. Thus, a systems biology approach was utilized to define putative targets of diclofenac, related to inflammation and oxidative stress. The results for topological analysis identified a cluster of 44 nodes, where only CAT enzyme was found to be directly modulated by diclofenac. In particular, this modulation regulated several other cellular processes (GO analysis), including glyoxylate and dicarboxylate metabolism, hydrogen peroxide metabolism, and cellular respiration. Importantly, CAT was identified as an H-B node in this cluster as well as in the entire CP-PPI network. Further analysis showed that CAT, by itself, was a highly connected node, which controlled the flow of information within the network. According to the theory of graphs, pharmacological modulation on H-B might induce pathway-network perturbations [46]. In the present case, these were reflected by a reduction in CAT activity in the trained animals receiving diclofenac. It has been previously demonstrated that diclofenac treatment suppressed CAT activity in gills but increased GPx activity, probably as a compensatory mechanism to reduce the accumulation of H2O2 [47]. The present study also assessed the effect of diclofenac on the glutathione redox cycle in rat model. Similar to the results reported in gills, the trained group displayed higher values for the ratio of GSH/GSSG, GR, and GPx enzyme activities. These results corresponded to the upregulation of antioxidant defense system, a signaling pathway that counteracted the inflammatory pathways triggered by acute bouts of exhausting exercise [8,45,48] and related to the increase in IL-6 [40,49].

## 5. Conclusions

The present study confirmed that chronic exercise training program triggers tissue adaptation mechanisms, which further contribute to better performances in tasks as observed in time-to-exhaustion tests. It also induces minor tissue damages after acute exercise. In particular, these adaptation mechanisms involved an increase in the liver and plasma inflammatory responses, such as cytokine production and release, and induction of oxidative stress. Further, the study highlighted that diclofenac treatment, which was aimed to reduce pain and provide a fast recovery after acute physical exercise, might result in a loss in exercise performance and tissue adaptations in the long term, mediated by blockage of the inflammatory/oxidative stress processes. Future studies are required to validate and identify the mechanisms responsible for the relevant role of CAT enzyme, which was identified using systems biology analysis.

## Figures and Tables

**Table 1:** Effect of training and diclofenac treatment on plasma biochemical parameters.

	SED-SAL	SED-DICLO	EXE-SAL	EXE-DICLO
Glucose (mmol/L)	5.81 ± 0.29	5.02 ± 0.19	5.06 ± 0.56	4.98 ± 0.33
Triglycerides (U/L)	2.47 ± 0.24a	1.81 ± 0.11a	1.20 ± 0.15b	1.54 ± 0.24b
ALT (mmol/L)	58.5 ± 3.54	60.11 ± 2.03	58.38 ± 1.85	56.18 ± 1.35
AST (mmol/L)	90.02 ± 3.20	91.02 ± 2.42	87.11 ± 1.22	89.49 ± 2.94
ALP (U/L)	231.1 ± 17.3	203.8 ± 17.3	207.1 ± 21.5	217.4 ± 15.7
dsDNA (fluorescence)	23.74 ± 5.53	19.5 ± 1.14	19.61 ± 2.29	20.73 ± 3.66

## Data Availability

Data is contained within the article.
